# Virological and clinical characterizations of respiratory infections in hospitalized children

**DOI:** 10.1186/1824-7288-39-22

**Published:** 2013-03-27

**Authors:** Suat Bicer, Tuba Giray, Defne Çöl, Gülay Çiler Erdağ, Ayça Vitrinel, Yesim Gürol, Gülden Çelik, Çigdem Kaspar, Öznur Küçük

**Affiliations:** 1 Yeditepe University, Faculty of Medicine, Department of Child Health and Diseases, Yeditepe University Hospital, Devlet Yolu Ankara Cad. No: 102-104,, Istanbul, Ataşehir, 34752, Turkey; 2 Yeditepe University, Faculty of Medicine, Department of Medical Microbiology, Yeditepe University Hospital, Devlet Yolu Ankara Cad. No: 102-104,, Istanbul, Ataşehir, 34752, Turkey; 3 Yeditepe University, Faculty of Medicine, Department of Medical Statistics, Yeditepe University Hospital, Devlet Yolu Ankara Cad. No: 102-104, Istanbul, Ataşehir, 34752, Turkey

**Keywords:** Child, Hospitalization, PCR, Respiratory infections, Viral respiratory pathogens, Respiratory viruses

## Abstract

**Background:**

The purpose of this study was to determine the incidence and seasonal distribution of viral etiological agents and to compare their clinical manifestations and disease severity, including single and co infections.

**Methods:**

Multiplex reverse-transcription PCR was performed for the detection of viruses in nasopharyngeal aspirat. Disease severity was grouped using a categorization index as very mild/mild, and moderate/severe. Clinical and laboratory characteristics of hospitalized children with viral respiratory tract infection were analyzed.

**Results:**

Viral pathogens were detected in 103/155 (66.5%) of patients. In order of frequency, identified pathogens were respiratory syncytial virus (32.0%), adenovirus (26.2%), parainfluenza viruses type 1–4 (19.4%), rhinovirus (18.4%), influenza A and B (12.6%), human metapneumovirus (12.6%), coronavirus (2.9%), and bocavirus (0.9%). Coinfections were present in 21 samples. Most of the children had very mild (38.8%) and mild disease (37.9%). Severity of illness was not worse with coinfections. The most common discharge diagnoses were "URTI" with or without LRTI/asthma (n=58). Most viruses exhibited strong seasonal patterns. Leukocytosis (22.2%) and neutrophilia (36.6%) were most commonly detected in patients with adenovirus and rhinovirus (p<0.05). Monocytosis was the most remarkable finding in the patients (n=48, 53.3%), especially in patients with adenovirus (p<0.05).

**Conclusions:**

RSV and RhV were associated with higher severity of illness in hospitalized children. RSV found to account for half of LRTI hospitalizations. In AdV and FluA and B infections, fever lasted longer than in other viruses. Coinfections were detected in 21 of the patients. The presence of coinfections was not associated with increased disease severity.

## Introduction

Acute respiratory tract infections (RTIs) are the most commonly encountered diseases for hospitalization, particularly in infants and young children. These are the major causes of morbidity and mortality with a worldwide disease burden estimated at 112 900 000 and 3.5 million deaths, respectively [1]. RTIs are divided into two categories, as acute lower respiratory tract infections (LRTIs) and upper respiratory tract infections (URTIs) according to localization of the infection. LRTIs are more harmful than URTIs. URTIs refer to infections of the nasopharynx, larynx, tonsillae, sinuses, ears, including rhinosinusitis, tonsillitis, pharyngitis, laryngitis/laryngotracheitis and otitis media. LRTI refers to infections of the trachea, bronchus, and alveolus, including tracheitis, bronchitis, bronchiolitis, and bronchopneumonia. The etiological agents include many bacteria, viruses and fungi. However, viral agents have a greater role in causing RTI as they are more commonly observed. In recent years, many viruses causing RTI have been discovered, including coronaviruses, human metapneumovirus and human bocavirus. Detection of these viruses relies on the polymerase chain reaction (PCR) in virology laboratories. As the agents of RTIs, viruses have constantly been shown to predominate; this majority of the viral agents may be related to the increased use of the PCR in the detection of viruses. Coinfections with more than one viral etiological agent exist in 4-33% of children [2,3]. The clinical severity of RTIs which are caused by viruses are viewed differently among viral agents. Coinfection may or may not be a risk factor for disease severity [4-6]. Evidence has also shown that severe viral infections in infancy and early childhood are related to recurrent wheezing and asthma in later life [7].

The purpose of current study was to determine the incidence and seasonal patterns of viral etiological agents and to compare their clinical manifestations and disease severity, including single and coinfections.

## Methods

### Patients, demographical factors, and clinical manifestations of acute RTIs

We have prepared a retrospective cohort study of RTIs in children, requiring hospitalization at a university hospital in Istanbul, Turkey. Children hospitalized with upper and/or lower respiratory tract symptoms and/or signs of RTI between November 2010 and September 2011, were eligible. The inclusion criteria for this study were: 1) children <9 years of age; 2) hospitalization at the pediatric ward of the Yeditepe University Hospital, between November 1, 2010 and September 30, 2011; and 3) a diagnosis of acute RTI, including URTI, acute bronchitis, acute bronchiolitis, and bronchopneumonia. The diagnosis for each patient was performed by the attending physicians based on standard clinical criteria. Bronchiolitis was recognised in patients <24 months in whom lower respiratory symptoms of wheezing, tachypnea and signs of respiratory distress such as nasal flaring, intercostal/subcostal retractions, and central cyanosis were observed. The diagnosis of bronchopneumonia was established on clinical findings which include fever, tachypnea and respiratory distress where focal or diffuse crackles or decreased vesicular sounds and radiographic findings such as infiltration and/or atelectasia, and/or air bronchograms were present. The diagnosis of URTI (including laryngotracheitis) was established on the basis of symptoms such as coryza, cough, earache, fever, sore throat, headache, and stridor.

Symptoms used for disease severity assesment were fever (tympanic temperature of ≥38.0°C), dyspnea, tachypnea, tachycardia (defined as heart rate above the 97th percentile for that age), hypotension (defined as blood pressure below the 3rd percentile for age), hypoxemia (defined as oxygen saturation in room air <94%), dehydration, and convulsion. Disease severity was assessed by using a categorization index which was similar to categorization of disease (viral RTIs) severity by Bezerra et al. [8], as follows:

1. Disease severity Group I: Very mild and/or mild disease. Very mild disease was described as the upper respiratory tract symptoms/signs only, requiring hospital admission and none or only one of the clinical symptoms/signs. Mild disease was described as the lower respiratory symptoms/signs +/2 upper respiratory tract symptoms/signs, requiring hospitalization and none or only one of the clinical symptoms/signs, and/or bronchiolitis score of 0–3 points.

2. Disease severity Group II: Moderate and severe disease. Moderate disease was described as the lower respiratory tract symptoms/signs +/2 upper respiratory symptoms/signs, requiring hospitalization and 2 of the clinical symptoms/signs, and/or the bronchiolitis score of 4–8 points. Severe disease was described as the lower respiratory symptoms/signs +/2 upper respiratory symptoms/signs, needing hospitalization and ≥3 of the clinical symptoms/signs, and/or the bronchiolitis score of 9–12 points.

Baseline vital signs including fever, oxygen saturation, respiratory rate, and pulse rate were listed at the time of assessment in the pediatric emergency department and daily afterward following admission in all patients. Patients with at least two symptoms and/or signs of RTI including fever, cough, nasal congestion, rhinorrhea, nasal flaring, intercostal/subcostal retractions, and central cyanosis, received a posterior nasal swab for virus detection, generally within five days of symptom onset. A sensitive multiplex PCR panel was used to demonstrate 14 viral agents during acute RTIs in the present study.

We assessed medical records of the patients for underlying diseases, past medical history, outpatient respiratory visits during the month prior to hospitalization, respiratory hospitalizations throughout the year prior to hospital admission, and recent respiratory symptoms of patients and other family members. The discharge diagnoses were divided into groups as “primarily URTI” (without LRTI and/or asthmatic attack) and “primarily LRTI” (with or without URTI and/or asthmatic attack).

### Nasopharyngeal aspirate (NPA) and PCR assays for respiratory pathogens

Fourteen respiratory viruses were included in the panel. Multiplex PCR was performed to demonstrate adenovirus (AdV), bocavirus (BcV), human coronaviruses (CoV) Group 1 (229E and NL63) and Group 2 (OC43 and HKU1), influenza viruses A&B (FluA&B), human metapneumovirus (MPV), parainfluenzaviruses type 1–4 (PIV1–4), respiratory syncytial virus (RSV), rhinovirus (RhV). We obtained a nasopharyngeal aspirate (NPA) sample using a sterile feeding tube and injector. NPA fluid samples were obtained more commonly by a nurse or sometimes a research assistant on all subjects within 48 hours of admission using a standardised protocol [8]. Samples were frozen at −20°C and transported on ice to the Department of Clinical Microbiology and Virology Laboratory of our university for viral nucleic acid amplification.

### Laboratory analysis

Bacterial blood cultures were obtained from 37 patients. Three to five milliliters of serum was obtained from each subject for complete blood counts, C-reactive protein (CRP) and erytrocyte sedimentation rate (ESR) analyses. CRP was evaluated as normal (<2.8 mg/L), mildly elevated (2.8-15 mg/L), moderately elevated (16–50 mg/L) and excessively elevated (>50 mg/L) in 91 children. ESR was evaluated in 60 children.

Cytopenia was determined with decreases in the three main cell groups which are leukocytes, platelets, and erythrocytes. Decreases in leukocytes and leukocyte subgroups (lymphocytes, granulocytes) were also analyzed according to age [9].

### Statistical analysis

The Fisher Exact Test was used to examine the differences in pathogen prevalence between disease severity categories I and II and/or different symptoms/signs; by setting the level of significance at p<0.05 as significant. Differences in seasonal distribution, demographic and clinical characteristics between viruses were examined using chi-square test, setting the level of significance at p<0.05 as significant. Statistical computations were done using SPSS 18 statistical package (SPSS Inc., Chicago).

For the purposes of analysis the following viral agents were grouped together: AdV, BcV, CoV, Flu A&B, PIV 1–4, RSV, and RhV.

## Results

### Demographical characteristics

One hundred-three patients under nine years of age hospitalized at the Pediatrics Department were enrolled. There were 61 males and 42 females with a male/female ratio of 1.4 to 1. The characteristics of patients are shown in Table 1. The mean age of patients was 34.9 (1–106) months, where 89.4% of patients where 5 years of age. Among the patients 18.4% were <12 months; 32% were 13–24 months of age, 39% were 25–60 months, and 10.6% were 61–106 months (Table 1). There were no statistically significant differences among age groups.

**Table 1:** The distribution of viral agents according to age

	**Total N (%)**	**Mean age (month)**	**Range (month)**	**0-12 months**	**13-24 months**	**25-60 months**	**61-106 months**
AdV	27 (26.2)	49.4	11-96	1	5	15*	6*
BoV	1 (0.95)	60	60	0	0	1	0
CoV	3 (2.9)	65	48-96	0	0	2	1
FluA&B	13 (12.6)	43.9	7-106	2	2	7	2
MPV	13 (12.6)	30.6	6-60	3	3	7	0
PIV1-4	20 (19.4)	32.6	1-106	4	6	9	1
RhV	19 (18.4)	26	7-72	3	11	4	1
RSV	33 (32)	26.5	3-104	9	13	9	2
***Total***	103 (100)	34.9	1-106	20	32	40	11

*: p<0.05.

There were significant difference for AdV between the the first group (0–12 months) with 3rd (25–60 months) and 4rd (61–106 months) groups.

Among the patients, 42 had other significant comorbidities (e.g. chronic bronchitis, other chronic diseases and prematurity). Thirty-four patients (33%) were attending school and daycare centers.

## PCR results

Viral respiratory pathogens were detected in 103/155 (66.5%) NPAs. The distribution of viral etiologic agents according to age is shown in Table 1. The detected pathogens were RSV (32%), AdV (26.2%), PIV 1–4 (19.4%), RhV (18.4%), Flu (12.6%), MPV (12.6%), CoV (2.9%), and BcV (0.9%), respectively. RSV was identified more than any other virus (33/103, p<0.01). Only three CoV and one BcV infections were detected. AdV was also detected frequently (26.2% of RTIs). Parainfluenza viruses were detected in 19.4% of patients with PIV3 manifesting the major part of PIV (10.6%). Influenza viruses were found in 12.6% of samples with Flu A being the most common (8.7%). The most common pathogens in single infections (n=82; 79.6% total samples) were RSV (28%) and AdV (24.4%) with CoV and BcV comprising a much smaller percentage (2.4% and 1.2% respectively) (Table 2). Coinfection with two or more pathogens were present in 21 samples (20.6%). Two pathogens were detected in 17 samples (16.5%), three pathogens in three samples (2.9%), and four pathogens were found in one sample (a two-year old boy with RSV, MPV, PIV2, and Flu A, who was hospitalized for URTI). The most common pathogens in coinfected samples were RSV (47.6%), RhV (47.6%), PIV (38%), AdV (33.3%), MPV (28.6%), Flu A&B (23.8%), and CoV (4.7%) where the most frequently detected coinfections were RSV/PIV (28.3% of coinfections) and RhV/AdV (19%) (Table 2).

**Table 2:** The distribution of viral etiologic agents and coinfections

	**Total (n)**	**%**	**Single infection**	**% in all single infections**	**Coinfection**	**% in all coinfections**
AdV	27	26.2	20	24.5	7	33.3
BoV	1	0.9	1	1.2	0	0
CoV	3	2.9	2	2.4	1	4.7
FluA&B	13	12.6	8	9.7	5	23.8
MPV	13	12.6	7	8.5	6	28.6
PIV1-4	20	19.4	12	14.7	8	38.0
RhV	19	18.5	9	11	10	47.6
RSV	33*	32.0	23	28	10	47.6
***Total***	103		82		21	

*: p<0.05.

Patients positive for BcV and CoV were significantly older (mean 60 and 65 months, respectively) than other hospitalized patients (mean 33.7 months). Patients positive for RhV and RSV were significantly younger (mean 26 and 26.5 months, respectively) than other hospitalized patients (mean 42.6 months) (p<0.05); although patients with MPV and PIV had similar mean age (30.6 and 32.6 months, respectively) (Table 1).

### Clinical manifestations and clinical diagnoses associated with viruses

Patients were classified under the three major clinical presentation groups (URTI only, URTI + LRTI, and LRTI only). Bronchopneumonia and URTI comprised the majority of discharge diagnoses. Overall, 58 had symptoms of URTI (56.3%) and 62 had symptoms of LRTI (60.2%), and 17 of these had symptoms of URTI and LRTI (16.5%). The discharge diagnoses of patients were divided as “URTI only” (39.8%), URTI + LRTI (16.5%), and “LRTI only” (43.7%). Among LRTI and URTI + LRTI patients, the discharge diagnoses were bronchiolitis (15.5%), bronchopneumonia (36%) and asthmatic attack (8.7%). Three of the children were hospitalized in the intensive care unit and one intubated. The distribution of discharge diagnoses are shown in Table 3.

**Table 3:** The distribution of clinical diagnoses

	**n**	**%**	**URTI only**	**URTI + LRTI**	**LRTI only**	Bronchiolitis*	Pneumonia*	Asthma*
AdV	27	26.2	22†	5	0	1	4	0
BoV	1		0	0	1	0	1	0
CoV	3		2	1	0	0	0	1
FluA&B	13	12.6	6	2	5	1	4	2
MPV	13	12.6	3	3	7	2	7	1
PIV1-4	20	19.4	7	6	7	1	11	1
RhV	19	18.5	10	3	6	1	6	2
RSV	33	32.0	6	5	22†	10	14	3
Coinfection	21	20.4	11	7	3	0	9	1
Single	82	79.6	30	10	42	16	28	8
TOTAL	103		41	17	45	16 (15.5%)	37 (36%)	9 (8.7%)

*: These patients are subgroups of LRTI. †: P<0.05.

The discharge diagnoses of patients were divided as “URTI only” (39.8%), URTI + LRTI (16.5%), and “LRTI only” (43.7%). Among LRTI and URTI + LRTI patients, the discharge diagnoses were bronchiolitis (15.5%), bronchopneumonia (36%) and asthmatic attack (8.7%).

Some viruses were more commonly identified with symptoms of URTI or LRTI (Table 3). Especially AdV infection was more commonly associated with URTI than LRTI (URTI: 81.5%, LRTI: 8.5%) (53.6% of cases of URTI vs. 8% of cases of LRTI; p=0.000) and RSV was more largely associated with LRTI (81.8%) than URTI (18.2%) (43.5% of LRTIs vs. 14.6% of URTIs; p=0.000). The number of patients in whom coinfection was found, was not different statistically in two major clinical manifestation groups (26.8% of URTIs; 16.1% of LRTIs).

### Severity of disease

Patients were divided into two groups according to level of clinical severity; 76.7% (n=79) of the patients were to be in the “disease severity Group I”, and 23.3% (n=24) of the patients were to be in the “disease severity Group II”. Patients who were in Group I have very mild (38.8%) and mild disease (37.9%), however, patients who were in Group II have moderate (14.6%) and severe (8.7%) disease. All of the patients with AdV were in Group I, they have mostly very mild disease than mild disease (81.5% vs. 18.5%, respectively), none of them had moderate and severe disease (p=0.000). Most of the patients with coinfection had very mild (47.6% of coinfections) and mild disease (38.0% of coinfections). Severity of illness was not worse with coinfections (Table 4).

**Table 4:** Correlation between disease severity and symptoms, laboratory/lung radiography results, hospitalization time, diagnosis groups

**Symptoms & laboratory & lung radiography results & hospitalization time & diagnosis groups**	**Disease severity**		
	**Group I**	**Group II**	**P**
AdV	27 (34.2%)	0	0.000
Flu A & B	11 (14%)	2 (8.3%)	NS
MPV	10 (12.6%)	3 (12.5%)	NS
PIV1-4	16 (20.2%)	4 (16.7%)	NS
RhV	12 (15.2%)	7 (29.2%)	NS
RSV	22 (27.8%)	11 (45.9%)	NS
Coinfection	18 (22.8%)	3 (12.5%)	NS
Single infection	61 (77.2%)	21 (87.5%)	NS
Leukocytosis	15 (19%)	5 (20.8%)	NS
Leukopenia	5 (6.3%)	0	NS
Positive lung radiography	20 (25.3%)	14 (58.4%)	NS
Hospitalization <48 hours	17 (21.5%)	0	0.01
Hospitalization 48–72 hours	29 (36.7%)	6 (25%)	NS
Hospitalization 73–96 hours	17 (21.5%)	4 (16.7%)	NS
Hospitalization 97–120 hours	8 (10.1%)	3 (12.5%)	NS
Hospitalization >120 hours	6 (7.6%)	11 (45.9%)	0.000
Hospitalization >96 hours	20 (25.2%)	15 (62.5%)	0.001
Primarily URTI	41 (51.9%)	0	0.000
Primarily LRTI	38 (48.1%)	24 (100%)	0.000
Bronchiolitis	11 (14%)	5 (20.8%)	NS
Bronchopneumonia	25 (31.6%)	12 (50%)	NS
Asthmatic attack	2 (2.5%)	7 (29.2%)	0.000

NS: Nonsignificant.

RSV was the second virus that caused hospitalization of young children. Patients positive for RSV (mean 26.5 months, 0–24 months: 66.7%) were significantly younger than the other hospitalized patients except for RhV (mean 26.0 months, 0–24 months: 73.7%). Hospitalized children with RSV had relatively a long hospitalization duration (>96 hours, 37.1%) compared to patients with other respiratory viruses.

Patients infected with RSV, MPV, and PIV1-4 were commonly diagnosed as LRTI (p<0.05), however, a higher percentage of AdV positive patients were diagnosed as URTI (p=0.000) in comparison to other viruses. Symptoms of hospitalized children were fever (50.5%), dyspnea (26.2%), tachypnea (24.2%), dehydration (12.6%), hypoxia (4.8%), hypotension (2.9%), and convulsion (3.8%). Clinical characteristics associated with viral pathogens are shown in Table 5 which displays clinical characteristics of single viral infections.

**Table 5:** Distribution of severe symptoms among single infections

**Single infections**	**AdV**	**FluA&B**	**MPV**	**PIV1-4**	**RhV**	**RSV**	**Total**
Fever >38.5°C	18* (90% in single AdV)	5	2	4	2	9	41
Dyspnea	0*	2*	3	3	4	10 (43.5% in single RSV)	23
Tachypnea	1*	1	3	3	4*	8	21
Hypoxia	0	1	0	1	2	1	4
Dehydration	3	1	1	2	2	2	11
Hypotension	0	1	0	0	0	1	2
Convulsion	1	0	0	0	1	0	2
***Total***	20	8	7	12	9	23	82

*: p<0.05.

Fever was observed in 50.5% of the patients with RTIs, and its duration was between 72–150 hours. This symptom was observed in 92.6% with AdV. Moreover, patients with single AdV infections were more likely to have fever than viral RTIs without AdV (p=0.000). AdV and influenza A&B had the longest mean duration of fever in RTI cases, 111.1 hours and 150 hours, respectively. The mean duration of fever in single viral infections was prolonged in patients with AdV (5.1 days). The rate of dyspnea in RTIs with single RSV infections was 92.6% (43.5% of dyspneic patients). Dyspnea was more likely to be detected with single RSV infections (43.5% of dyspneic patients).

### Seasonal distribution

**Figure 1:**
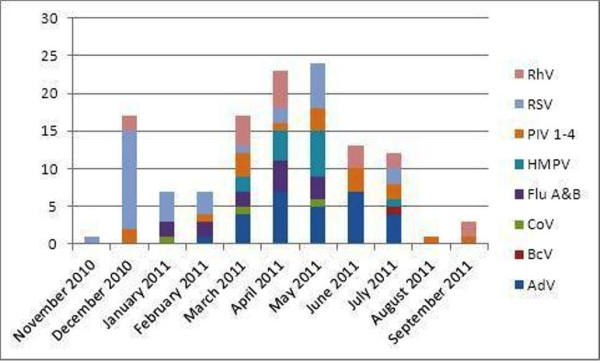
The seasonal distribution of respiratory viral agents.

The seasonal distribution of viruses is displayed in Figure 1. Most viruses exhibited strong seasonal patterns. Some viruses were most frequently detected in the cold and rainy season. The clear seasonal patterns of influenza A&B and CoV (winter/spring), RSV (fall/winter/spring) and MPV (spring) somewhat overlapped in terms of incidence. Influenza A was identified during January through May with a peak occurrence in April. Influenza B was observed in January/March/May. BcV and some cases of ADV were determined in the warm months. PIV and RhV did not have a clear seasonal pattern. RhV was detected throughout the study months, although its percentage rose in the spring (March and April), and decreased between December-March. Different PIVs (PIV1-4) were identified in different months during the year the study was conducted. PIV1 was identified in March and June, PIV2 in February and May, PIV4 in June and August, while PIV3 predominated during March, and continued through May with a peak in December. CoV and BcV were detected in low percentages during the study period.

### Laboratory results

White blood cell counts were available for 90 (87.4%) of the patients. Leukocytosis was detected in 20 (22.2%) patients. Especially AdV (n=10) and RhV (n=6) were more commonly associated with leukocytosis (p<0.05). Neutrophilia was detected in 33 patients (36.6%), especially with AdV (n=15, p=0.000) and RhV (n=9, p=0.013). Monocytosis was the most significant finding in the patients (n=48, 53.3%) (p<0.05). Nearly all of the viruses were associated with monocytosis, especially in the AdV (n=16, p<0.05) patients. Neutropenia was detected in 10 patients, especially in those with RSV (n=4) and PIV (n=4). Lymphopenia was detected in 19 patients which was significant between 2 and 8 years of ages (p<0.05). Nearly all of the viruses were associated with lymphopenia; however, this was not a statistically significant difference among viruses. During the 4-week follow-up period, white blood cell counts returned to normal together with the improvement of clinical symptoms. None of the patients had permanent cytopenia. The differences in haematological alterations between the single and coinfection groups were statistically not significant.

CRP and ESR were detected in 91 (88.3%) and 60 (58.2%) patients, respectively. High CRP values were the most remarkable finding in the patients. CRP values were found to be mildly (n=21), moderately (n=29) and excessively (n=21) elevated in 71 patients; however, it was not elevated in 21 patients. ESR was elevated in 44 patients; however, it was not elevated in 16 patients. Blood culture was studied in 37 (35.9%) of the patients. None of them had positive blood cultures.

## Discussion

The gold standard for diagnosing respiratory viruses is a viral culture of nasopharyngeal samples and/or throat samples. However, the process may require 3–7 days, which is a long duration for diagnosis. Although viral culture is the “golden standard” of diagnosing virus infection, it limits the sensitivity of the diagnosis, as conventional viral culture has a low sensitivity in comparison to RT-PCR. Nucleic acid PCR types of laboratory tests with short durations (of less than 24 hours) have become available and with high sensitivity. This study clearly demonstrates the clinical manifestations, clinical severities and seasonal patterns of respiratory viral agents of URTI and LRTI in Turkish children. Furthermore, it provides new information on the epidemiology of viral agents in children by assessing the significance of RSV, MPV, RhV, AdV, and the newly identified viruses (PIV4, CoV, BcV). Viruses were detected in 66.5% of the hospitalized patients. This detection rate was similar to those found in other studies of hospitalized patients (35-90%) [3,10-12].

Coinfections were common, occurring in 20.4% of RTIs, with some pathogens, such as RhV and MPV, being commonly identified as coinfections while others, such as AdV and RSV were mostly detected as single infections. Similar rates (between 11.1% and 39.6%) were found in other PCR based studies [10,11,13,14]. Sensitive RT-PCR assays make it possible to identify a large spectrum of viral agents, but an identified virus may be concerned with persistence of a virus after a recently afflicted RTI. RhV and BcV may persist in the upper airways for a long duration. Up to two months of persistence has been shown in nasopharynx [14-17]. AdV shedding with the faeces may persist for a long time after an acute infection [18]; however, extended shedding of AdV in the nasopharynx was not documented. For this reason, this persistence also may not occur in the nasopharynx. However, identification of two or more viruses in a patient may be due to prolonged viral shedding or asymptomatic persistence of viruses and further work would be needed to clarify this situation. Identification of the viral genome quantitively may be helpful in explaining coinfections.

Evidence pertaining to the severity of coinfections compared to single viral infections is conflicting. In our study, coinfections were not more severe than single infections. The presence of coinfections was not associated with extended duration of fever. The severity of coinfection may depend on the viruses involved. In contrast with some previous studies [4,19,20], coinfections were not more severe than single infections in this study. Other studies conducted do support this finding [5,6]. This may have resulted due to smaller numbers of patients in the moderate and severe categories.

Very few studies have included the full spectrum of disease severity from mild to life threatening severity. In this study, over 75% of the patients had a mild or moderate disease. Most viruses were detected in all disease severity groups. However, patients with RSV and RhV had moderate and severe disease symptoms more frequently and patients with AdV, CoV and BcV were did not. These viruses were not the cause of moderate and severe symptoms in our patients. RSV, RhV and MPV were also more likely to be detected in patients with bronchiolitis and bronchopneumonia, confirming and highlighting the importance of these pathogens.

Our study has estimated the incidence of respiratory viruses among the hospitalized children with the diagnosis of RTI that may be considered as viral etiology. It has also demonstrated the importance of RSV and AdV among LRTIs and URTIs, respectively. RSV was the most significant viral agent associated with pediatric hospitalizations for LRTI (32%), especially in patients under two years of age, similar to findings in other studies (12–60%) [6,12,17]. It was detected in a larger proportion (35.5%) of hospitalized young (<24 months) patients. RSV was identified in 43.5% of patients with pneumonia. RSV is the second virus that results in a longer hospitalization duration (>96 hours; 37.1%) in young children (<24 months) compared to other viruses. It was especially evident in young children that, RSV was the most frequently detected virus in this group. Similar to other studies, RSV was associated with higher severity of diseases. Fever (36.3%), dyspnea (36.3%) and tachypnea (30.3%) were the most commonly encountered symptoms in hospitalized children with RSV. It was detected during November through July with peaks in December and May. In 47.6% of coinfected patients, RSV was identified together RhV, AdV, PIV, FluA&B and MPV.

PIV is the second most common virus in our study that associated with LRTI hospitalization (21% of LRTI). It was detected in a larger proportion of hospitalized patients with RTI (19.4%) than in other similar LRTI studies (range 0.4–9%) [6,17,20]. This finding was consistent with 1990–2004 United States National Respiratory and Enteric Virus Surveillance System data showing PIVs second only to RSV detected in patients <5 years of age with RTIs [21]. Singleton et al. detected PIV as a cause of LRTI in hospitalized children with respiratory diseases (18%) [12]. In our study, PIV3 caused a greater percentage (55%) of PIV infections. PIV was detected in a smaller percentage (16.1%) of hospitalized young children (<24 months) compared to RhV and RSV. Severity of infection in hospitalized children with PIV was mild and moderate in general compared to other viruses. Mean duration of fever is 111.6 hours in hospitalized children with PIV; this is relatively longer compared to other viruses except for influenza (150 hours). PIV is the third virus that results in a long hospitalization duration (>96 hours; 20%) after RSV and RhV. PIV1-4 were identified across all the studied months with peak the rate during early and late spring and early summer. PIV1-4 was detected with other viruses in 38% of the coinfected patients, especially with RSV.

RhV was the second most common agent identified in hospitalized children in our study. RhV is one of the increasingly identified viral agents with the use of PCR [22,23]. It is the most common cause of URTI (especially common cold) in both adults and children. RhV may also be associated with LRTI [24], and was detected in 14-24% of hospitalized children with LRTI [25,26]. RhV may now be considered a major cause of LRTIs in infants [27] and of asthma-triggering in children [28,29] and adults [30]. The role of RhV in asthma exacerbations was documented by Johnston et al.; it accounted for 65% of virus-induced asthmatic attacks [28]. Studies in recent years [14,31] have shown higher rates of viruses in children hospitalized for asthmatic attacks; however, studies in the later years [32] have shown lower rates (19% to 42%) compared to recent studies. The more extensive use of PCR for virus identification in recent years may be responsible for the higher rates in recent studies.

RhV has been identified as the single virus in some LRTIs [19,30], however, in this study more than half (52.6%) of the patients with RhV was identified in coinfections. This was not correlated with other studies. Hospitalized children with RhV were usually encountered during summer months [19,30]. The seasonal pattern of RhV in this study correlates with these studies; RhV was mostly encountered in spring and summer with a peak incidence occurring in March and April. The disease severity of patients with RhV was relatively higher compared to other viruses. Patients with RhV were 29.2% with moderate to severe symptoms. In children with RhV, tachypnea (42.1%), dyspnea (36.8%), fever (36.8%), and dehydration (21%) were encountered at levels of severity similar to those observed in children with RSV. RhV causes the second longest hospitalization duration (>96 hours; 25.7%), which is especially evident in young children (<24 months; 35%). RhV was identified with AdV, RSV, PIV, MPV and CoV in 47.6% of coinfected patients.

MPV is one of the causes of upper and lower RTIs, which is a well-recognized viral agent of pneumonia in preschool and older children, but not a frequently identified agent in children younger than the former two groups [3,33]. In contrast to some studies, MPV was mostly detected in young (<24 months) hospitalized children in our study. MPV was detected in a higher percentage (12.4%) of hospitalized children than in other studies (3–5%) [6,17] except for one study in Alaska (15%) [12]. MPV infection was associated with URTI and especially LRTI (77%) in hospitalized children. In contrast to Singleton et al. [12], MPV infection was associated with mild severity of disease in our study, but patients, especially young children, have a relatively long hospitalization time (>96 hours; 25%), compared to children with other viruses except RSV and RhV. MPV was identified during spring and summer with a peak in May. MPV was detected with other viruses in 28.6% of coinfected patients, especially with FluA&B, RSV and RhV.

FluA&B was detected in a relatively small percentage (12.6%) during winter and spring in our study. However, this percentage was higher than the ones reported in other studies (0.8–8%) [6,12,17,20]. FluA&B was identified during January through May with a peak in April. Seasonal patterns of FluA&B, CoV, RSV and MPV overlapped. Patients with FluA&B were older than patients who were diagnosed with bronchiolitis caused by other viruses. FluA&B infection was associated with LRTI (53.8%) and moderate disease severity in patients. Fever (53.8%) and dyspnea (30.8%) were the most commonly encountered symptoms in hospitalized children with FluA&B. Mean duration of fever for this virus is longer than other viruses (150 hours) in hospitalized children with FluA&B. In patients with H1N1 influenza A, fever was reporded in 94.6% of patients with a mean level of 38.6°C [34]. The mean duration of fever was 4.8 days in these patients, which was similar to that of our patients (4.63 days). Fever (100%) and other symptoms and signs, reported by Hançerli et al. [35] among hospitalized children in Istanbul included cough (82%), dyspnea (32%), vomiting (27%), myalgia (19%), headache (18%), rhinorhea (15%), diarrhea (13%), and sore throat (8%).

The clinical characterization of AdV infection is designated by high grade (>39°C) and prolonged (up to 120 hours) fever [36]. Bezerra et al. reported that, AdV was the second most common virus associated with RTI hospitalization [8]. Similar to other studies, AdV was also commonly identified but not associated with severe illness [8]. Hospitalized patients with AdV have symptoms of URTI (81.5%) and LRTI (18.5%). Fever (92.6%) was the most commonly encountered symptom in hospitalized children with AdV. The duration of fever in this infection was more prolonged (up to 120 hours) in our patients, compared to other viruses; this finding was similar to other studies [36]. Dehydration (11.1%), febrile convulsion (7.4%) and tachypnea (3.7%) were encountered rarely in these patients. Chen et al. reported that, fever was recorded for 98.7% of patients with AdV, and the mean level of fever was 39.2°C [37]. While the mean duration of fever was 4.8 days in their patients, our patients experienced a similar duration (4.63 days). Other symptoms and signs include cough (77.6%), rales (23.3%), diarrhea (25.2%), vomiting (21.8%), abdominal pain (18.9%), conjunctivitis (13.6%), rashes (7.9%), and febrile seizure (3.1%) as reported by Chen et al. [37]. The peak incidence of AdV occurred among children 2–5 years of age in our study, while Chen reported the peak to occur between 4 and 8 years of age in Taiwan [37]. Similar to our study, other studies reported that most AdV infections occurred among children under 5 years of age [36,38]. Children hospitalized with AdV were less likely to have LRTI, and were older than patients with other viruses. The mortality rates were reported in patients with AdV in Korean (19%) [38] and Argentinean (28.6%) [39] children. There was no mortality in patients with AdV, similar to the Chen report [38]. Our patients with AdV mostly had URTI, such as tonsillitis and pharyngitis (81.5%), with a few patients with LRTI (18.5%). In a national report prepared for 2006–2007, AdV was detected in 5.6% of children hospitalized with acute LRTI, but, clinical characteristics were not investigated [10]. Similar to the rate reported in this study viruses were detected in LRTI, but not URTI. AdV was shown in LRTI in our study group in 4.8% of patients. Furthermore, AdV was identified during February through July with peaks in April and June. AdV was detected with other viruses in 33% of coinfected patients, especially with RhV.

Viral infections are known to cause hematologic complications particularly cytopenies. Leukocytosis may also occur in viral infections, as well as bacterial infections. Lymphopenia was the most common form of cytopenia that was seen in viral infections, particularly with FluA&B [40,41]. Lymphopenia was the most notable finding in our cases with FluA&B in children two years or older. The main hematologic manifestation of influenza is lymphopenia with decreased lymphocyte/neutrophil ratio. Leukocytosis and neutrophilia were detected in the patients with AdV and RhV. Monocytosis was the most significant finding in the patients with AdV. There was not any negative effect on the hematologic conditions of coinfections.

## Conclusion

The importance of respiratory viruses as the etiologic agent of RTIs in hospitalized children was verified in our study. Furthermore, RSV, AdV, PIV and RhV were detected in over two-thirds of hospitalized patients with RTI. RSV was found to account for half of LRTI hospitalizations. PIV, MPV and RhV were associated with LRTI. Our study summarizes the clinical characteristics of viral agents that were detected in children with RTI. The severity of diseases caused by RSV and RhV were higher than other viruses. In AdV and FluA&B infections, fever lasted longer than in other viruses. Coinfections were detected in 21% of the patients, especially with some pathogens, such as RhV and MPV, whereas, AdV and RSV were more commonly identified as single infections. The presence of coinfections was not associated with increased disease severity. RSV, Flu A&B, AdV, CoV and MPV exhibited strong seasonal patterns, with Flu A&B and MPV cases peaking over the rainy season (January-May and March-May, respectively), and AdV peaking soon afterwards (April-July). RhV and PIV were endemic throughout the year, although RhV activity did appear to decrease between December-March and RSV activity did appear to decrease after May. Different PIVs circulated at different times of the studied year.

## Abbreviations

(RTIs): Acute respiratory tract infections; (LRTIs): Lower respiratory tract infections; (URTIs): Upper respiratory tract infections; (PCR): Polymerase chain reaction; (NPA): Nasopharyngeal aspirate; (AdV): Adenovirus; (BcV): Bocavirus; (CoV): Human coronaviruses; (FluA&B): Influenza viruses A&B; (MPV): Human metapneumovirus; (PIV1–4): Parainfluenzaviruses type 1–4; (RSV): Respiratory syncytial virus; (RhV): Rhinovirus; PCR: Polymerase chain reaction; CRP: C-reactive protein; (ESR): Erytrocyte sedimentation rate.

## Competing interests

The author(s) declare that they have no competing interests.

## Authors’ contributions

SB conceived of the study, and participated in its design and coordination and drafted the manuscript. TG participated in the design of the study, and collection and acquisition of data. DÇ helped to the design of the study, collection and acquisition of data, and helped to draft the manuscript. GÇE helped to collection and acquisition of data, and general supervision of the research group. AV involved acquisition of funding, general supervision of the research group, coordination and helped to draft the manuscript. YG and GÇ carried out molecular analysis. ÇK performed the statistical analysis. ÖK conceived of the study, participated in its design and helped to draft the manuscript. All authors read and approved the final manuscript.
